# Limited usefulness of the IS*6110* touchdown-PCR in blood for tuberculin skin test false-negative cattle with serological response to *Mycobacterium bovis*

**DOI:** 10.3389/fvets.2024.1359205

**Published:** 2024-05-20

**Authors:** Micaela Encinas, Ximena Ferrara Muñiz, Romina Ayelén Sammarruco, Victoria Ruiz Menna, Carlos Javier Garro, Fernando Delgado, Analía Macías, Gabriel Magnano, Martín José Zumárraga, Sergio Gabriel Garbaccio, María Emilia Eirin

**Affiliations:** ^1^Instituto de Agrobiotecnología y Biología Molecular (IABiMo) UEDD CONICET-INTA, Centro de Investigación en Ciencias Veterinarias y Agronómicas (CICVyA)-CNIA, Hurlingham, Argentina; ^2^Instituto de Patobiología Veterinaria (IPVET), UEDD CONICET-INTA, Instituto Nacional de Tecnología Agropecuaria (INTA), INTA-CONICET, Hurlingham, Argentina; ^3^Departamento de Patología Animal, Facultad de Agronomía y Veterinaria, Universidad Nacional de Río Cuarto, Río Cuarto, Argentina

**Keywords:** bovine tuberculosis, anergy, diagnosis, IS*6110* touchdown-PCR, blood, false-negative, tuberculin skin test

## Abstract

Ante-mortem diagnosis of bovine tuberculosis (bTB) is based mainly on the tuberculin skin test (TST) and the ɣ-IFN release assay (IGRA). Some infected animals escape screening tests, thus, limit herd sanitation. Previous reports have suggested a predominant pattern of multi-organ lesions attributable to *Mycobacterium bovis* (the causative agent of bTB) bacteraemia. A case–control study was conducted to investigate blood PCR as an alternative tool for improving ante-mortem detection of TST false-negative bovines. Cases comprised 70 TST false-negative bovines (cases), which were serology positive, and controls included 81 TST positive bovines; all of them confirmed as infected with *M. bovis*. Detection of the IS*6110* target through touchdown blood-PCR (IS*6110* TD-PCR) was performed. The positivity of the blood-PCR was 27.2% in the control group. This performance was similar to the 15% obtained among cases (*p* = 0.134). Most cases identified by the IS*6110* TD-PCR exhibited focalized lesions (*p* = 0.002). Results demonstrated that blood-PCR could detect TST false-negative cattle, even if they are negative for IGRA. Considering that cases exhibited humoral response to *M. bovis*, further studies conducted in a pre-serological stage could provide evidence about the real contribution of the technique in herds.

## Introduction

Bovine tuberculosis (bTB), a chronic disease disseminated worldwide, impacts on the agricultural countries. Its etiological agent, *Mycobacterium bovis*, infects a wide range of mammalians, including humans. The control and eradication programs of this disease consist of a “test and slaughter” policy by using the *in vivo* tuberculin skin test (TST) as the primary screening tool (1). The TST is highly effective but has limitations. The diagnosis maybe compromised by high rates of exposure to non-tuberculous mycobacteria (NTM) (2), and the existence of non-reactors to the screening tests. Previous reports showed the presence of false-negative animals to the screening tests in endemic herds (3–10).

TST false-negative bovines constitute a challenge for herd sanitation since they remain in herds as a source of infection. In Argentina, Garbaccio et al. (5) reported that 76% of TST false-negative dairy cattle were also negative to the IGRA, which suggests that tests based on cellular-mediated immune response partially detect the infection among these animals. In addition, humoral response detects TST false-negative cattle from endemic herds, which is more likely associated to late stages of the disease (4, 5, 10). However, some TST and IGRA false-negative animals could be in a pre-serological stage and therefore, they would not be detected in herds.

TST false-negative cattle presented exacerbated gross lesions with a predominant multi-organ commitment (5), which can occur because of haematogenous dissemination of the bacilli from the primary infection area. This fact suggests that viable mycobacteria could be detectable in blood samples, as previously confirmed in naturally infected cattle (11). Regarding *M. bovis* detection in blood, previous studies developed different specific PCRs in blood, targeting *M. bovis* sequences (*mpb70*, *esxB* gene, RD4 region, IS*6110*), with variable results (12–16).

Zumárraga et al. (17) developed a touchdown cycling for PCR, with the IS*6110* element as target (IS*6110* TD-PCR), which is highly specific for the *Mycobacterium tuberculosis* complex (MTC) members (17). IS*6110* TD-PCR showed an enhanced sensitivity compared to the standard IS*6110*-PCR and detected *M. bovis* DNA in different biological samples (tissues, milk, nasal swabs) and colony cultures (colony-PCR), for direct diagnosis in infected *M. bovis* bovines (17–20). However, the usefulness of IS*6110* TD-PCR in blood samples remains unexplored.

The objective of the study was to evaluate the performance of the IS*6110* TD-PCR in TST false-negative cattle from bTB endemic herds. Results confirm the presence of *M. bovis* DNA in blood, and constitute evidence for future studies to evaluate the usefulness of the technique in a pre-serological stage of infection.

## Materials and methods

### Study design

A case–control study was conducted to assess the performance of the IS6*110* TD PCR in blood of Friesian Holstein bovines (*n* = 151), predominantly older than 2 years old, with confirmed bTB. Animals came from 31 different dairy herds under sanitation programs located in the central productive dairy region of Argentina.

The control group (*n* = 81) included bovines positive to the TST according to the Argentinean official guidelines. Selection of cases (*n* = 70) was made among non-reactors to the TST, considering those animals positive for humoral response against *M. bovis* by Enzyme-Linked Immunosorbent Assay (ELISA) (5). Blood from cases and controls was tested by PCR.

Cases were also tested by IGRA, as an ancillary test to the TST that also evidences cell-mediated immune response.

The pathology pattern was evaluated at the slaughterhouse inspection to characterize the disease presentation among cases. It was recorded as one organ with lesions or more than one organ with lesions (multi-organ lesions).

Animals were bTB confirmed when they were positive for at least one of the following tests: bacteriology, histopathology-ZN staining and tissue-PCR.

### Tuberculin skin test

Accredited veterinarians performed the caudal fold TST (*CF*-TST) according to official guidelines of the Service for National Agri-Food Health and Quality (Law 128/12)^1^. Because the *CF*-TST was applied in herds under sanitation, an animal exhibiting a skin-fold thickness increase ≥3 mm was positive (severe interpretation test).

### Interferon-gamma release assay (IGRA)

All cases were tested by IGRA. Heparinized blood (200 μL) from the jugular vein was stimulated with 25 μL of the following antigens: Avian Tuberculin PPD 2500 (250 IU/mL), Bovine Tuberculin PPD 3000 (300 IU/mL), sterile Phosphate Buffered saline (PBS) solution 1X and BOVIGAM® Pokeweed Mitogen (5 μg/mL) in a 96-well cell culture plate. Harvested plasma were stored at −20°C for γ-IFN measurement by ELISA using a commercial bovine γ-IFN-microplate ELISA. Blood stimulation, ELISA as well as interpretation of data (standard interpretation, cut-off = 0.1) were performed according to the manufacturer’s instructions (BOVIGAM TB kit, Thermofisher).

### Detection of humoral response by ELISA

Humoral ELISA tested sera of non-reactor TST animals. Blood (10 mL) was obtained from the jugular vein 15–20 days’ after *CF*-TST. Serum (100 μL, 1,100 in PBS 1X solution), was centrifuged at 2500 *g* and then used to test antibody against bovine protein purified derivative (PPDB). ELISA consisted of a binding step (12 μL of PPDB per well, 1 μg/mL) performed over night at 4°C, a washing step (five times with a solution of PBS 1X-Tween 0.5, 1% of skimmed milk) and an incubation step with sera (100 μL, diluted 1,100 in PBS 1X solution) 1 h at 37°C. The plate was washed with PBS 1X and 100 μL of conjugate anti-Bovine IgG−Peroxidase antibody (Sigma, dilution 1:7000) was added to incubate the samples with this secondary antibody for 1 h at 37°C was added. The plates were washed five times more. Finally, 100 μL of the developing substrate (Citrate Buffer + ABTS (Sigma) + H_2_O_2_) were added and incubated for 10 min in the dark. The reaction reading was performed at 405 nm. Optical densities (OD) ≥ 0.42 classified animals as positive (5).

### Blood-PCR

Blood (10 mL) anticoagulated with EDTA (0.5 mg/mL) was obtained from the jugular vein. DNA extraction was performed with a commercial kit (PuriPrep-S kit, InbioHighway), following the manufacturer’s instructions. As blood-PCR is not a routine protocol performed in our laboratory, some evaluations were performed before testing the presence of *M. bovis* DNA in the samples. The quality (A_260_nm/A_280_nm ratio) and concentration (A_260_) were assessed by spectrophotometry (NanodropTM, Thermo Fisher Scientist). The DNA integrity was also analyzed by an electrophoresis in a 0.8% agarose gel (TAE buffer 1X, 80 volts for 60 min.) stained with 5 μg/mL of ethidium bromide (Promega, USA). Amplification of a 450 bp fragment of the endogenous *16S* mitochondrial ribosomal RNA gene (*16S*RNArmt) (21) was also performed by using GoTaq® G2 DNA Polymerase (Promega, USA). The amplification products were visualized by electrophoresis in a 1.2% agarose gel (TAE buffer 1X, 90 volts for 45 min) stained with ethidium bromide, as described above, and with a 100 bp molecular weight marker (100 bp Plus DNA Ladder, Trans, China).

The presence of *M. bovis* DNA in blood was assessed using an IS*6110* specific MCT fragment (IS*6110* TD-PCR) using the HotStarTaq® Master Mix kit (Qiagen, USA), as described previously (17). The products were visualized by electrophoresis in a 2% agarose gel (TAE buffer 1X, 80 volts for 45 min) stained with ethidium bromide, as described above, and with a 100 bp molecular weight marker (100 bp Plus DNA Ladder, Trans, China). The expected amplification product was of 245 bp.

### Post-mortem inspection and sampling

Official veterinarians performed a detailed inspection searching for lesions compatible with bTB, according to the official slaughterhouse procedures. Sampling of representative portions of the retropharyngeal and submandibular lymph nodes (LN), respiratory (tracheobronchial and mediastinal LN, and lung), digestive (mesenteric and hepatic LN, and liver), and mammary (udder and supra mammary) LN were aseptically collected in individual containers to perform tissue-PCR and bacteriology. In addition, other pieces were disposed in containers with 10% buffered formalin for fixing tissues for histopathology. Regarding cases, after performing the pathology inspection, animals were classified as those exhibiting one organ with macroscopic lesions or those with multi-organ lesions.

### Bacteriology and histopathology

For bacteriology, 30 g of each tissue was cut into small pieces with scissors and put into a sterile bag with 20 mL of double-distilled sterile water. Maceration was performed for 3 min. (Basic Masticator, IUL Instruments type 470, Spain) and the homogenate was decontaminated by Petroff’s method (22). An aliquot of 2 mL of each decontaminated sample was inoculated on egg-based Stonebrink solid media at 37°C with biweekly observation, for at least 8 weeks. This inoculation was done in triplicate. Suspected *M. bovis* colonies were stained with Ziehl-Neelsen (ZN) to identify acid-fast bacteria (23). For histopathology, fixed samples were dehydrated with different alcohol solutions of increasing strength (50, 70, 80, 95% and absolute ethanol), subsequently clarified with xylene, and finally, embedded in paraffin. The paraffin plugs were cut in 5 μm-thick sections (Leica RM2125 RTS, Biosystems), deparaffinized, hydrated and stained with hematoxylin-eosin and ZN staining (23).

### Tissue-PCR

Two different PCR were interchangeably used to check the presence of *M. bovis* DNA in the collected tissues: IS*6110* TD-PCR (17), with an expected product of 245 bp, and an adapted touchdown-PCR from a previously described Rv*2807* nested-PCR (Rv*2807* TD-PCR) (24). DNA from tissues was obtained using a commercial extraction kit (ADN PuriPrep-T Kit, InbioHighway, Argentina) according to the manufacturer’s instructions. GoTaq® G2 DNA Polymerase (Promega, USA) was used according to the manufacturer’s instructions. Briefly, the adaptation step of the Rv*2807* TD-PCR consisted of an initial denaturation at 96°C for 3 min, with eight cycles of 96°C for 1 min, with an annealing temperature that was gradually reduced from 72°C to 64°C, and an extension step at 72°C for 1 min. The procedure was followed by 30 cycles including a denaturation step at 96°C for 1 min, annealing at 66°C for 1 min, extension at 72°C for 1 min 45 s, and a final extension at 72°C for 8 min. The expected amplification product was of 443 bp. PCR products were visualized in an 2% agarose gel (TAE buffer 1X), 80 volts for 45 min, stained with 5 μg/mL of ethidium bromide (Promega, USA), and compared to a 100 bp molecular weight marker (100 bp Plus DNA Ladder, Trans, China). Image digitalization was performed in a Geldoc Genetic Analyzer (Bio-Rad Laboratories).

### Statistical analysis

The statistical comparison of proportions was performed with EpiDat 3.0 version software (Xunta de Galicia, OPS-OMS). Calculated *p* values equal to or less than 0.05 were considered statistically significant. The DNA concentration and quality were analyzed to identify significant outliers (*p* > 0.05) using the Grubbs’ test (25). Data distribution was tested by the Shapiro Wilk test with median and interquartile range of 25–75 (RIQ_25–75_) as descriptive statistics. Mean comparisons were performed using unpaired Student’s *t* test (GraphPad Software, Inc.). The Fisher’s exact Test was used to evaluate the association between the IS*6110* TD-PCR results in blood and the pathology profile.

## Results

The *in vitro* IGRA test, that represents an additional tool that measures cell-mediated immune response, recorded 55.4% of negativity among cases. This result shows that animals either positive or negative to this ancillary screening test, were similarly represented within the *CF*-TST false negative bovines (*p* > 0.05) (Table 1).

**Table 1:** Performance of the blood IS*6110* TD-PCR in *CF*-TST false-negative bovines.

	Ante-mortem		Post-mortem		
	IGRA		Macroscopic lesions		Confirmation**
	+	–	One organ	Multi-organ	
Blood IS6*110* TD-PCR + 15.7% (*n* = 11)	33.3% (3/9)	66.7% (6/9)	60% (6/10)	40% (4/10)	72.3% (8/11)
Blood IS6*110* TD-PCR – 84.3% (*n* = 59)	46,4% (26/56)	53.6% (30/56)	12,1% (7/58)	87,9% (51/58)	93.2% (55/59)
Total* (*N* = 70)	44.6% (29/65)	55.4% (36/65)	19.1% (13/68)	80.9% (55/68)	90% (63/70)

Shows the performance detection, as the percentage (% = (n/N)*100), of the blood-PCR targeting the IS*6110* specific sequence as well as the ante-mortem and post-mortem results obtained in *CF*-TST false-negative cattle. *Sample size analyzed for each technique was variable. **Animals were confirmed for bTB when exhibited a positive result in almost one of the following confirmation tools: bacteriology, histopathology-ZN staining and tissue-PCR.

At the slaughterhouse inspection, cases exhibited 97.1% of macroscopic lesions compatible with bTB. Of these animals, 80.9% developed multi-organ lesions and, in a lesser proportion, 19.1% of them granulomas limited to one organ. Two animals lacked macroscopic lesions, although with a positive result of histopathology-ZN staining (Table 1).

DNA extraction from blood was performed in all the studied bovines (*n* = 151). A median genomic DNA concentration of the cases was 5.9 (3.6; 11.2) ng/μL, significantly lower than that obtained in the control group, in which the median concentration was 27.0 (19.0; 56.3) ng/μL (*p* < 0.0001). Despite this difference, the quality of the templates according to A_260nm_/A_280nm_ ratio and the positive results for the *16S*RNArmt PCR suggested good conditions of the extracted DNA (Supplementary Figure 1). Thus, the template was useful for further downstream determinations such as the IS*6110* TD-PCR.

Blood-PCR performed in cases and controls (*n* = 151), yielded 22% of positivity for the IS*6110* TD-PCR. Data stratification revealed that 27.2% of controls were positive for the IS*6110* TD-PCR, while a lower proportion (15.7%) of the cases was positive for this test; however, the differences were not significant (*p* = 0.134).

On the other hand, IS*6110* TD-PCR yielded significantly higher positive results in animals with focused lesions (46.2%), compared to animals with multi-organ lesions (7.3%) (*p* = 0.002). In addition, 66.7% of the IS*6110* TD-PCR positive samples were IGRA negative (Table 1).

## Discussion

Bovine tuberculosis, a health concern for animals and humans, is an ancient disease distributed worldwide, ranks among the top zoonotic threats (26). Currently, TST false-negative cattle constitute a challenge for diagnosis, because these animals remain in herds spreading the disease. Some of these bovines also fails to react to ancillary diagnosis tools such as the IGRA (3, 5).

In Argentina, our group reported cases of these TST false-negative bovines with repeatedly negative results for the *CF*-TST and a predominant multi-organ pathology in herds under sanitation, most of these animals were negative for IGRA (5). In Spain, TST and IGRA double negative bovines were identify positive for bacteriology. Researchers reported that these animals yielded a significant higher proportion of positive culture in beef cattle compared to dairy cattle (3).

In experimental infections*, M. bovis* promotes a predominance of cell-mediated immunity but with low or absent antibodies during the initial stages. The humoral response increases as the infection progresses, which is associated with the exacerbation of the disease pathology (27, 28). TST false-negative cattle exhibit humoral response, and previous studies demonstrated that antibody detection against *M. bovis* could contribute to identify these animals in naturally endemic herds (5, 6, 10). However, the association of antibodies with later stages of the disease could limit the performance of serology-based tools to detect those false-negative bovines that have not yet reached this humoral stage.

The present study constitutes valuable evidence to understand how to deal with this phenotype in endemic bTB herds. However, it is important to highlight that the results obtained here are biased given the selection criteria to identify *CF*-TST false-negative bovines in endemic herds. This selection was based on the presence of humoral response. Some questions remain open: Can the double TST and IGRA false-negative cattle be identified before the humoral stage? Are there false-negative animals with lesions but no humoral response, despite a putative advanced infection stage?

*M. bovis* genome detection in blood samples constitutes an approach poorly developed among positive reactors in the literature, and with no antecedents among the *CF*-TST false-negative phenotype. In Ethiopia, PCR targeting the *M. bovis mpb70* gene in blood samples detected a significantly higher proportion (68%) of positive animals (12) than the obtained in the present research (*p* < 0.0001). These animals belonged to bovines chronically infected, as screened by the cervical TST. In our study, *CF*-TST positive bovines came from herds under sanitation, were highly infected bovines are not expected to be present for an extensive period. These differences could limit the sensitivity of IS*6110* TD-PCR in blood samples. Conversely, a previous Indian study reported no IS*6110*-PCR positive blood samples from cervical-TST positive cattle with confirmed infection (14). The researchers used the phenol-chloroform-isoamyl alcohol method to extract DNA, in contrast to the present study and the research of Elsohaby et al., who used optimized commercial kits. Thakur et al. did not perform control checks on the quantity and quality of the extracted DNA, which are essential to ensure the usefulness of a DNA sample for downstream applications, including PCR (29).

By performing the IS*6110* TD-PCR in milk of bovines, Zumárraga et al. (20) demonstrated the presence of *M. bovis* in bTB free herds of a dairy productive area with hot spots of infection in Santa Fe province, Argentina. Based on this study, the authorities incorporated the detection of *M. bovis* in dairy herds as a surveillance complementary tool in the Regional Plan for the control and eradication of bovine tuberculosis in Santa Fe province (Law 949/12, Ministry of Production of Santa Fe province, Argentina). However, the results of the present study suggest that the use of PCR for blood samples could be useful, not only for dairy cattle, but also for beef cattle, and in every period of the productive cycle.

Despite the predominant multi-organ lesions observed among the *CF*-TST false-negative bovines, this pathology pattern would not necessary reflect a greater presence of mycobacterial DNA in blood.

A possible explanation would be that circulating naked DNA, viable bacilli associated with bacteraemia or both, could be present in blood but in an intermittent manner.

*CF*-TST false-negative bovines limit the sanitation of bTB infected herds based on the application of ante-mortem screening tests, supporting the search of improved diagnosis tools. Results presented here, although preliminary, confirm the presence of *M. bovis* DNA in bovine blood samples of the *CF*-TST false-negative bovines suggesting the useful of this technique to be considered. However, further studies conducted in a pre-serological stage could provide more evidence about the real contribution of the technique in herds.

## Data availability statement

The raw data supporting the conclusions of this article will be made available by the authors, without undue reservation.

## Ethics statement

The animal studies were approved by CICUAE Instituto Nacional de Tecnología Agropecuaria, CICVyA. The studies were conducted in accordance with the local legislation and institutional requirements. Written informed consent was not obtained from the owners for the participation of their animals in this study because Study was performed in endemic herds. Animals detected as *M. bovis* infected must be sent to the slaughterhouse due to the infection in Argentina is a mandatory reportable disease.

## Author contributions

MEn: Data curation, Formal analysis, Investigation, Methodology, Writing – original draft, Writing – review & editing. XF: Data curation, Methodology, Writing – review & editing. RS: Data curation, Investigation, Methodology, Writing – review & editing. VR: Data curation, Methodology, Writing – review & editing. CG: Data curation, Methodology, Writing – review & editing. FD: Data curation, Investigation, Methodology, Writing – review & editing. AM: Investigation, Methodology, Writing – review & editing. GM: Investigation, Methodology, Writing – review & editing. MZ: Conceptualization, Funding acquisition, Resources, Supervision, Writing – review & editing. SG: Conceptualization, Data curation, Formal analysis, Funding acquisition, Project administration, Resources, Supervision, Writing – review & editing. MEi: Conceptualization, Data curation, Formal analysis, Funding acquisition, Methodology, Project administration, Resources, Supervision, Writing – original draft, Writing – review & editing.

## Supplementary material

FIGURE S1 Features of DNA templates and IS6*110* TD-PCR in CF-TST false-negative bovines. **(A)** DNA concentration and quality. Scatter plot of concentration (ng/μL) and Quality (proportion A260/A280) of DNA from blood samples from CF-TST false-negative bovines (*n* = 70) was determined by spectrophotometry. Outliers, previously identified by Grubbs test (GraphPad), were excluded from the statistical analysis. **(B)** DNA integrity templates obtained from bovine blood samples. Electrophoresis in a 0.8% agarose gel stained with ethidium bromide to evaluate DNA integrity and RNA contamination of samples (loading sample: 5 μL). S1–S9 refers to nine different genomic DNAs extracted from blood samples belonging to CF-TST false-negative bovines. “High molecular weight” represent the region of the gel expected to show DNA not disrupted, as a dense band of high molecular genomic DNA. “Degradation” shows the region of the gel in which genomic DNA degradation, observed as a continued and diffuse band, it is visualized, and finally, the “RNA contamination” indicates the area of the gel in which contaminating RNA that has co-eluted with the genomic DNA during the extraction process is expected to be detected. **(C)** IS6*110* TD-PCR amplification in bovine blood samples. Electrophoresis in a 2% agarose gel to visualize the amplification of IS6*110* target in blood samples of CF-TST false-negative bovines. Red arrow indicates a band of 245 bp corresponding to IS6*110* amplification sequence. S1–S9 refers to DNA extracted from different animals. PC, positive control (*M. bovis* DNA, AN5 strain). CC, contamination control (DNase and RNase free water); MWM, molecular weight marker.
