# Supplementary figures and images for: Limited usefulness of the IS6110 touchdown-PCR in blood for tuberculin skin test false-negative cattle with serological response to Mycobacterium bovis
Source: Front Vet Sci. 2024 May 20;11:1359205. doi: 10.3389/fvets.2024.1359205 (PMC11149419; doi:10.3389/fvets.2024.1359205)

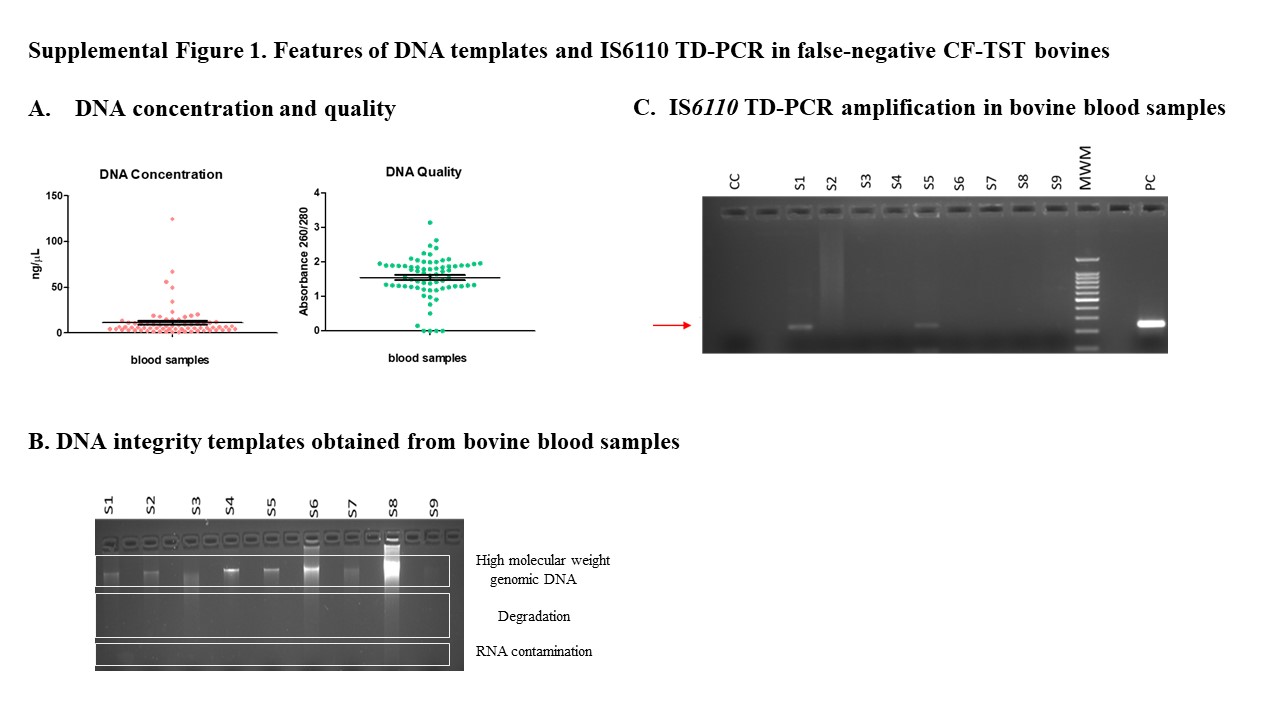

Supplement: FIGURE S1 — Features of DNA templates and IS6110 TD-PCR in CF-TST false-negative bovines. (A) DNA concentration and quality. Scatter plot of concentration (ng/μL) and Quality (proportion A260/A280) of DNA from blood samples from CF-TST false-negative bovines (n = 70) was determined by spectrophotometry. Outliers, previously identified by Grubbs test (GraphPad), were excluded from the statistical analysis. (B) DNA integrity templates obtained from bovine blood samples. Electrophoresis in a 0.8% agarose gel stained with ethidium bromide to evaluate DNA integrity and RNA contamination of samples (loading sample: 5 μL). S1–S9 refers to nine different genomic DNAs extracted from blood samples belonging to CF-TST false-negative bovines. “High molecular weight” represent the region of the gel expected to show DNA not disrupted, as a dense band of high molecular genomic DNA. “Degradation” shows the region of the gel in which genomic DNA degradation, observed as a continued and diffuse band, it is visualized, and finally, the “RNA contamination” indicates the area of the gel in which contaminating RNA that has co-eluted with the genomic DNA during the extraction process is expected to be detected. (C) IS6110 TD-PCR amplification in bovine blood samples. Electrophoresis in a 2% agarose gel to visualize the amplification of IS6110 target in blood samples of CF-TST false-negative bovines. Red arrow indicates a band of 245 bp corresponding to IS6110 amplification sequence. S1–S9 refers to DNA extracted from different animals. PC, positive control (M. bovis DNA, AN5 strain). CC, contamination control (DNase and RNase free water); MWM, molecular weight marker. [file Image_1.jpeg]
